# Current perspectives and trends in colorectal cancer and cancer-associated fibroblasts: a review and bibliometric analysis

**DOI:** 10.3389/fimmu.2025.1618742

**Published:** 2025-07-03

**Authors:** Chengyong Qian, Lingling Ren, Shuyuan Zhu, Fangling Chen, Haiping Shen, Xiangcheng Hu, Guanglan Chen

**Affiliations:** Digestive Endoscopy Center, The Second People’s Hospital of Lishui, Lishui, Zhejiang, China

**Keywords:** cancer-associated fibroblasts, colorectal cancer, bibliometrics, tumor microenvironment, trends

## Abstract

**Background:**

Cancer-associated fibrocytes (CAFs), a key component of the tumour microenvironment, are marked by their heterogeneity. They also exhibit a high degree of plasticity. In the last two decades there has been a strong association established between CAFs and colorectal carcinoma (CRC). However, there are no comprehensive statistics on CRC or CAFs, and the potential directions for research.

**Methods:**

The study performed a literature review spanning from January 1, 2004, to March 27, 2025, within the Web of Science Core Collection Database. VOSviewer and CiteSpace software were used to perform bibliometric analysis and visualization. Microsoft Excel and R was also utilized.

**Results:**

The analysis included 1145 articles. The articles in question were published across 359 different journals, and included 4032 keywords. The number of publications increased significantly between 2010 and 2025. China was the leading contributor to the total number of publications, and the United States led the global list of citations. Sun Yat-sen University and Shanghai Jiao Tong University are renowned research institutions. Notable researchers such as De Wever, Olivier and Bracke, Marc from Ghent University Hospital, and Pena, Cristina from Puerta de Hierro Majadahonda University Hospital are among the most productive and highly cited authors. CANCERS has the most publications, and the highest citation rate. CAFs are a major focus of research in CRC. This includes the effect of CAFs, such as on cell proliferation and angiogenesis.

**Conclusion:**

This study uses bibliometric analyses to present a comprehensive view of research in CAFs, CRCs from 2004 until March 27, 2025. The study highlights important research areas, anticipates future directions and offers valuable insights to future efforts in the field.

## Introduction

The International Agency for Research on Cancer’s (IARC) 2022 Global Cancer Statistics released colorectal carcinoma (CRC), with an estimated incidence rate of 9.6%, ranks third among malignant tumors worldwide. Its mortality rate, however, is second among cancers and accounts for 9.3% global cancer deaths. Studies have also shown that the CRC affects younger populations. It is now the leading cause of mortality amongst young men in the United States, and second amongst young women, placing a heavy burden on the healthcare system and economy (1, 2). For CRC treatment, surgical intervention, chemotherapy and radiotherapy serve as the primary clinical approaches. Metastatic or advanced CRC has a poor prognosis, and both chemotherapy and radiotherapy are associated with significant side effects. Recent years have seen significant progress with CRC treatments. Investigational strategies for CRC treatment focus primarily on the targeting of driver genes, including EGFR mutations as well as BRAF, KRAS and MEK (3, 4). Due to the mutations of various immune evasion pathways, CRC metastasis and recurrence persist despite these advancements. Understanding the mechanisms of resistance is essential in order to combat drug resistance effectively. Developing novel immune or targeted therapeutics with better efficacy but fewer side-effects will be key for future CRC treatments (5).

In our continuing quest to understand cancer, increasing evidence suggests that tumor microenvironment complexity and diversity in organs and patient populations contributes to cancer progression and metastasis and also mediates tumor cell resistance to drugs. The TME is now a key area of cancer treatment and research. Cancer-associated fibrocytes (CAFs), spindle shaped, functionally active fibroblasts within the TME, which is a complex, dynamic response are of major concern (6). The significance of CAFs in the TME has been increasingly recognized through extensive research, which also reveals their connections to CRC progression, treatment resistance, and unfavorable patient prognoses (6–9). It is therefore essential to understand the role that CAFs play in CRC, to develop novel therapeutic approaches to overcome drug resistance.

Pritchard & Hawkins define bibliometrics in two ways: “application of mathematical and statistics methods to books, other media and communication” as well as “quantitative evaluation of bibliographic features of literature.” respectively (10).Researchers across disciplines increasingly use bibliometric tools to quickly grasp new research frontiers, focal points and areas of focus in certain fields (11, 12). A bibliometric study in this area may be a useful tool for analyzing research and improving awareness. It is therefore important to perform a bibliometric study of the CAFs’ role in CRC. Through this analysis, it is possible to pinpoint the focal points and emerging directions in the study of CAFs related to CRC.

This study aims to perform a bibliometric evaluation of the relationship between the CRCs and the CAFs. It is intended to assist both novices and experienced researchers in expanding their knowledge, finding new research areas and visualizing future plans.

## Materials and methods

### Database and search strategy

We chose the Web of Science Core Collection. The primary search term was “cancer-associated fibroblasts”, and the search was refined using information from previous studies (13, 14). The search strategy was described as follows: TS=(cancer-associated fibroblasts: “cancer-associated fibroblast*” OR “tumour-associated fibroblast*” OR “tumor-associated fibroblast*” OR “tumor associated fibroblast*” OR “tumour associated fibroblast*” OR “cancer associated fibroblast*” OR “tumor-related fibroblast*” OR “tumor related fibroblast*” OR “carcinoma-associated fibroblast*” OR “carcinoma associated fibroblast*” OR “tumor associate fibroblast*” OR “cancer-associated myofibroblast*” OR “cancer associated myofibroblast*” OR “tumor-associated myofibroblast*” OR “tumor associated myofibroblast*” OR “tumour-associated myofibroblast*” OR “tumour associated myofibroblast*” OR “tumour associate fibroblast*” OR “cancer associate fibroblast*” OR “carcinoma associate fibroblast*” OR “cancer-related fibroblast*” OR “cancer related fibroblast*” OR “tumour-related fibroblast*” OR “tumour related fibroblast*” OR “carcinoma-related fibroblast*” OR “carcinoma related fibroblast*”) AND TS=(“Rectal Neoplasm*” OR “Rectal Tumor*” OR “Rectal Cancer*” OR “Rectum Neoplasm*” OR “Rectum Cancer*” OR “Cancer of the Rectum” OR “Cancer of Rectum” OR “Colorectal Neoplasm*” OR “Colorectal Tumor*” OR “Colorectal Cancer*” OR “Colorectal Carcinoma*” OR “Colonic Neoplasm*” OR “Colon Neoplasm*” OR “Cancer of Colon” OR “Colon Cancer*” OR “Cancer of the Colon” OR “Colonic Cancer*”). This study spans a period of 20 years from 1 January 2004 to 27 March 2025. The study incorporates articles and reviews authored in English. We conducted thorough searches and screenings to minimize the potential bias that could be caused by database updates. We also took strict measures to deal with possible duplicate publications. We used EndNote to remove duplicates from data that was exported directly from WOSCC’s database. The software detects and removes duplicates by matching details such as titles, authors and publication dates, DOIs and journal names. The software ensured no duplicates or search strategies from the database entered into the analysis. Two researchers then manually checked deduplicated literature to ensure no duplicates were left. They examined titles, abstracts and full text. The manuscript was finally completed by April 16th, 2025.

### Data analysis

Microsoft Excel 2019 was used to create statistical tables, and fit curve analysis. We also created flowcharts using Microsoft Word 2019. We used the bibliometrix tool in R 4.3.1 to perform Lotka’s Law, and Bradford’s Law analysis. We visualized international collaborations between countries using an online bibliometric website (https://bibliometric.com/), and performed country visualization analyses with Origin 2024. VOSviewer and Citespace were used to perform bibliometric analyses of keywords, authors, institutions and journals. We focused on coauthorship, cooccurrence and cocitation patterns. The data was cleaned, the misspelled words were corrected manually, and we merged all overlapping elements into one element.

### Lotka’s law and Bradford’s law

Lotka’s Law describes, using power-law distributions as a basis, the relationship between productivity of authors and frequency of publication (15). There are far more authors with a single publication than those with numerous publications. Through distribution patterns, Lotka’s Law evaluates the inequality or concentration in a field while also quantifying the productivity of writers. Lotka’s law may be formulated in mathematical terms as follows (16):


$$A(n)=\frac{A(1)}{n^{2}}$$

In this equation, A(n) denotes the total number of papers published by n authors, whereas A(1) corresponds to the total articles written by a single author.

Bradford’s Law is a useful tool to help divide journals into zones. The journals fall into three groups. Zone 1 serves as the central area, encompassing the majority of journals. About 25% of journals are in Zone 2. The third zone includes journals that have only published a few articles on the subject (17).

## Result

**Figure 1:**
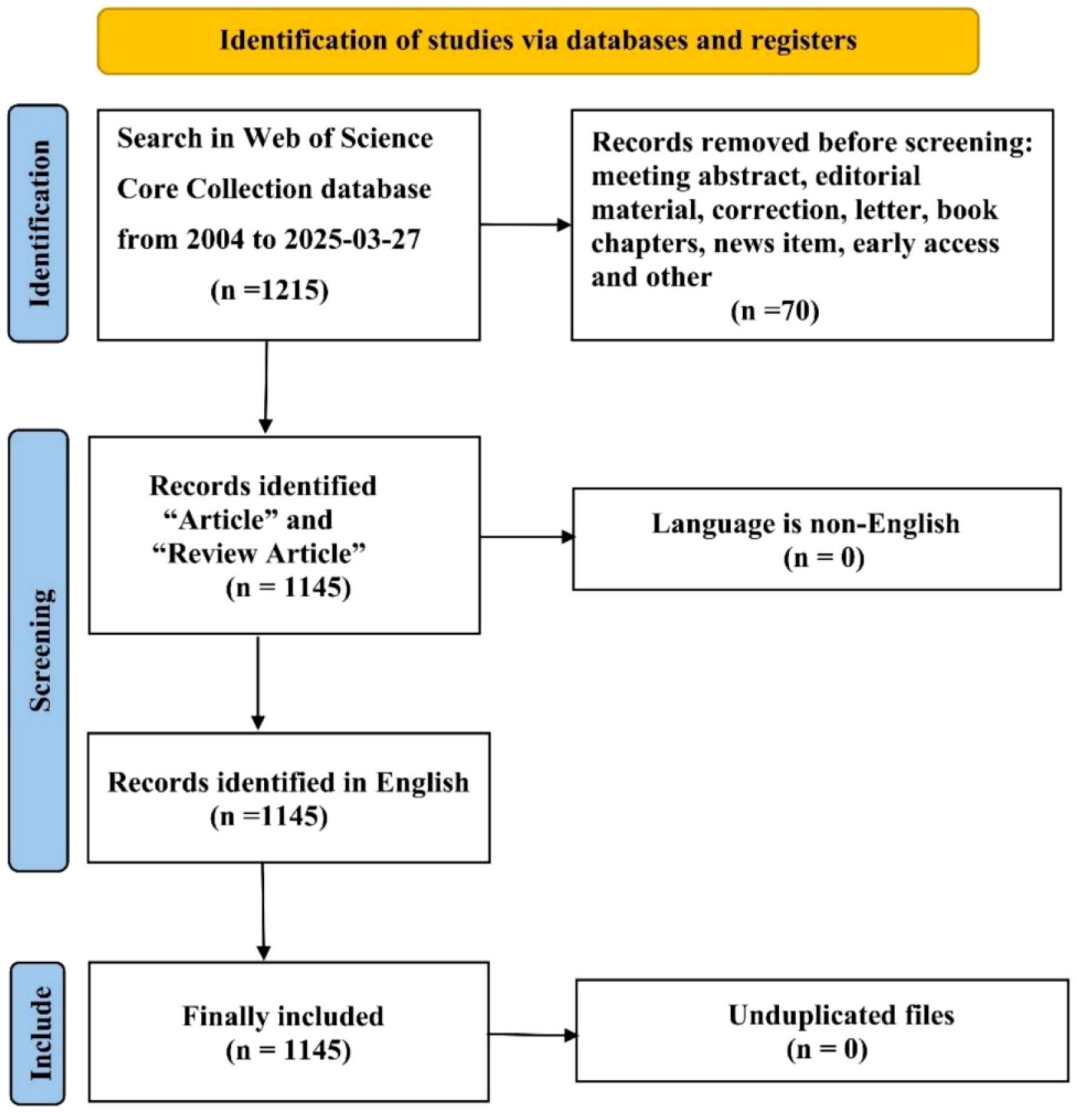
Flowchart illustrating the search strategy and exclusion criteria.

The WOSCC contained 1215 articles. We selected 1145 of the 1215 articles after establishing criteria for exclusion based upon publication date, type of article, and language. In **Figure 1**, we show the specific workflow.

### Analysis of the number of publications

**Figure 2:**
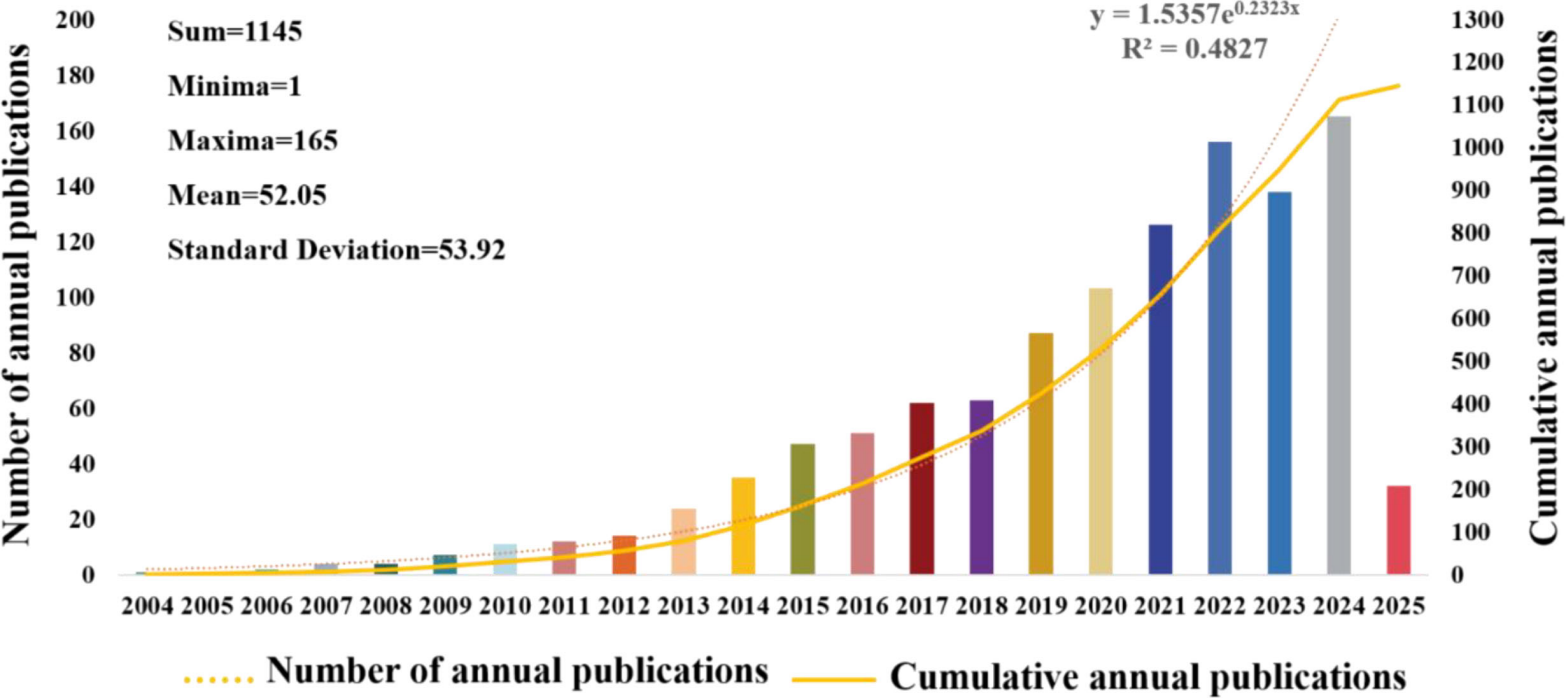
Graph illustrating the annual increase in publications regarding colorectal cancer and cancer-associated fibroblasts.

**Figure 2** illustrates the rising trend in annual publications related to CRCs and CAFs. This upward trend is expected to continue from 2004 through 27 March 2025. Between 2004 and 2009, the number of articles produced was minimal, with a total output of 19, showing a slow growth trend. From 2010 to 2013, the number of publications increased steadily to 61, doubling the output of the previous phase. Starting in 2014, there was a sustained and significant rise in publications, with the cumulative number reaching 1,145 by March 27, 2025. This accounts for roughly 94.7% of all articles published from 2004 to March 27, 2025, although there was a minor decline in 2023 relative to 2024. The fitting curve and related formulas suggest that research on CAFs and CRC is gaining increasing attention and has entered a phase of rapid development, becoming a popular research direction.

### Situation of countries/regions and institutions

**Figure 3:**
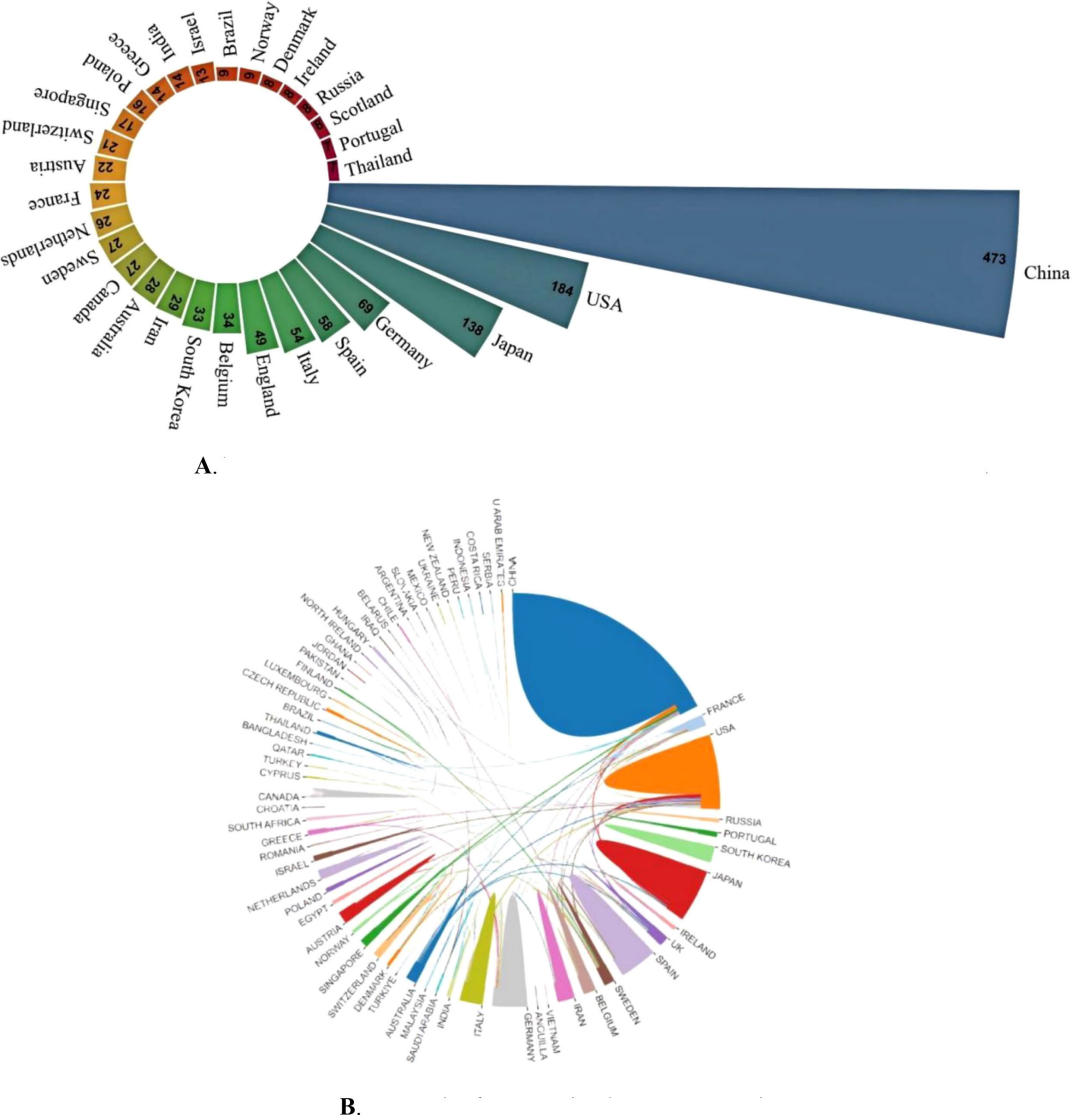
**(A)** Nightingale’s Rose diagram displaying publications from the top 30 countries. **(B)** Network of cooperation between countries.

In total, 65 countries published articles about CAFs and the CRC. In **Figure 3A** are the 30 countries with the highest productivity. China is the country with the most publications (N=473). The United States and Japan are next (N=184). In terms of citation frequency, the United States ranks first with 16,260 citations, ahead of China (N=14,921) and Japan (N=5,349). The high number of citations in the United States indicates that American scholars have greater academic influence, with more innovative and higher-quality papers that are widely recognized in the field. **Figure 3B** illustrates the international collaboration among different countries in CAFs and CRC research. The United States occupies a large area in the figure and has very thick lines connecting it to many other countries, indicating the strongest collaborative intensity in this research area. France also has thick lines connecting it to multiple countries, especially the United States, highlighting its significant role in international collaboration. Japan has thick lines connecting it to other countries, particularly the United States and France, indicating a high level of collaborative intensity. Despite having a lower intensity of international collaboration than the United States, France, and Japan, China has the highest publication output in CAFs and CRC research, highlighting its active research engagement in this field. With increasing scientific research investment and stronger international collaboration, China has the greatest potential for future development.

**Figure 4:**
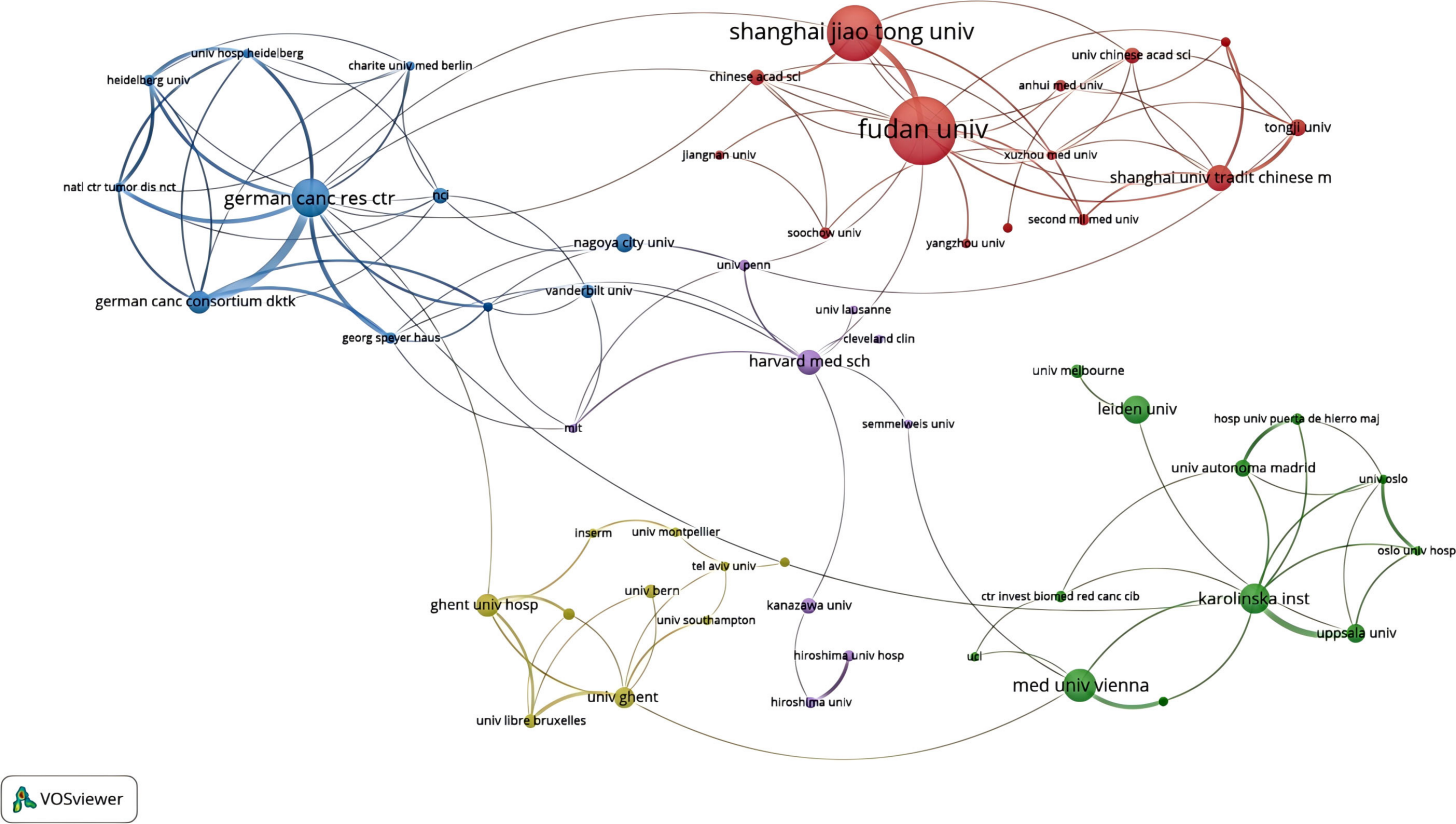
An institutional collaboration network map in the field of colorectal cancer and cancer-associated fibroblasts.

In the CRC field, 1,617 different institutions have published publications about CAFs. The publication rankings show Sun Yat-sen University at the top with 102 publications. Fudan University follows with 94, then Shanghai Jiao Tong University (N=58), Medical University of Vienna (N=56), and Nanjing Medical University (N=50). In **Table 1**, the top 20 universities have been ranked. Sun Yat-sen University is the university with the most publications. However, in terms of total and average counts of citations, it trails behind Fudan University and the Medical University of Vienna. **Figure 4**, a collaboration map of institutions, shows that 125 institutions have published more than five papers. In terms of link strength, the top five institutions are the German Cancer Research Center (total link strength of 44), Fudan University (N=40), Shanghai Jiao Tong University (N=31), Medical University of Vienna (N=18), and Karolinska Institute (N=16).

**Table 1:** The top 20 leading institutions in cancer-associated fibroblasts and colorectal cancer research.

Rank	Institution title	Records	Total citations	Average citation
1	Sun Yat-sen University	102	383	3.75
2	Fudan University	94	476	5.06
3	Shanghai Jiao Tong University	58	192	3.31
4	Medical University of Vienna	56	414	7.39
5	Nanjing Medical University	50	73	1.46
6	Zhejiang University	49	81	1.65
7	Taipei Medical University	47	60	1.28
8	Leiden University	37	489	13.22
9	Cent South University	37	144	3.89
10	Zhengzhou University	36	263	7.31
11	Sichuan University	35	180	5.14
12	Huazhong University of Science and Technology	33	311	9.42
13	Southern Med University	33	113	3.42
14	National Cancer Center Hospital East	30	106	3.53
15	German Cancer Research Center	28	202	7.21
16	Wuhan University	27	29	1.07
17	China Medical University	26	11	0.42
18	Nagoya University	25	325	13
19	Shandong University	25	117	4.68
20	Seoul National University	23	44	1.91

### Authors analysis

**Figure 5:**
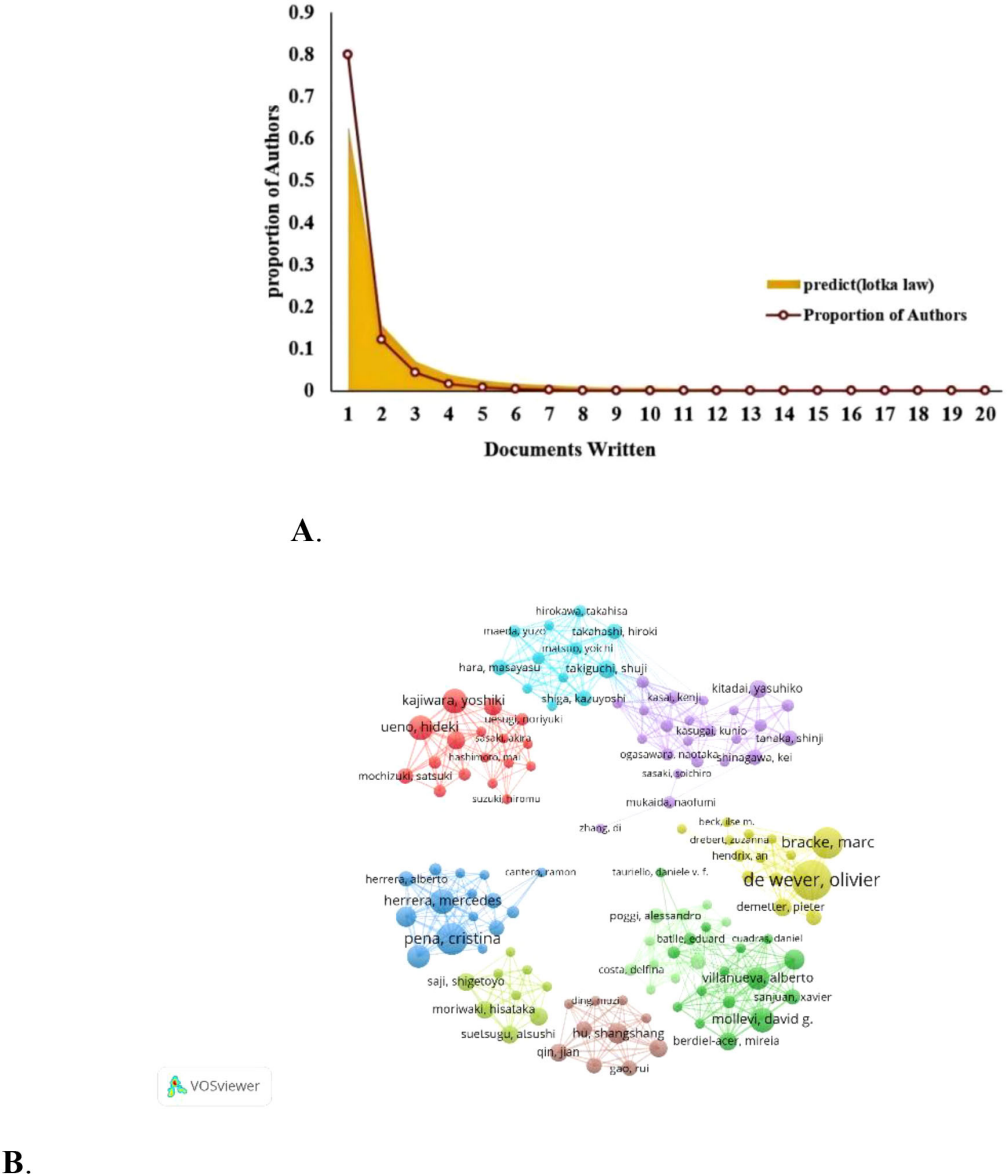
**(A)** Author productivity analysis according to Lotka’s law. **(B)** Researchers’ collaboration network map in colorectal cancer and cancer-associated fibroblasts studies.

7604 researchers have been involved in research related to CAFs or GC. Lotka’s Law, which measures scientific productivity in the field of science, shows that 80% of all authors have only published one article (**Figure 5A**). Only 12.1% of them have published more than two articles and 4.3% of them have published at least three. **Table 2** lists the top 20 authors with the highest productivity and citation counts in this field. Leading among them is De Wever, Olivier from Ghent University Hospital, with 16 publications. Close behind are Bracke, Marc from the same institution and Pena, Cristina from Puerta de Hierro Majadahonda University Hospital, each with 12 publications. These three authors also have the highest citation counts, underscoring their significant contributions and leadership in CAFs and CRC research. Additionally, Greten, Florian R. (9 publications, 808 citations), Herrera, Mercedes (9 publications, 716 citations), and Kajiwara, Yoshiki (9 publications, 194 citations) are notable researchers in the CAFs field. We also identified eight distinct author clusters with active collaboration between them (**Figure 5B**). De Wever and Olivier are the most closely co-operating authors, followed by Cristina Pena, Kajiwara Yoshiki Villanueva Alberto Kitadai Yasuhiko Moriwaki Hisataka Hu Shangshang Takiguchi Shuji. This highlights their important roles in this field. However, the extent of collaboration between interconnected nodes in distinct clusters is quite limited, which highlights the insufficiency in external communication.

**Table 2:** The 20 most productive and highly cited authors in cancer-associated fibroblasts and colorectal cancer research.

Rank	Authors	Counts	Total citations	Average citation
1	De Wever, Olivier	16	1409	88.06
2	Bracke, Marc	12	1224	102
3	Pena, Cristina	12	1189	99.08
4	Greten, Florian r.	9	808	89.78
5	Herrera, Mercedes	9	716	79.56
6	Kajiwara, Yoshiki	9	194	21.56
7	Mollevi, David g.	9	275	30.56
8	Ueno, Hideki	9	194	21.56
9	Zhang, Wei	9	100	11.11
10	Bonilla, Felix	8	864	108
11	Dolznig, Helmut	8	533	66.63
12	Villanueva, Alberto	8	262	32.75
13	Bergmann, Michael	7	176	25.14
14	Garcia De Herreros, Antonio	7	804	114.86
15	Hu, ShangShang	7	26	3.71
16	Ostman, Arne	7	407	58.14
17	Salazar, Ramon	7	252	36
18	Berdiel-acer, Mireia	6	245	40.83
19	Chen, Wei	6	70	11.67
20	Demetter, Pieter	6	930	155

### Journals analysis

**Figure 6:**
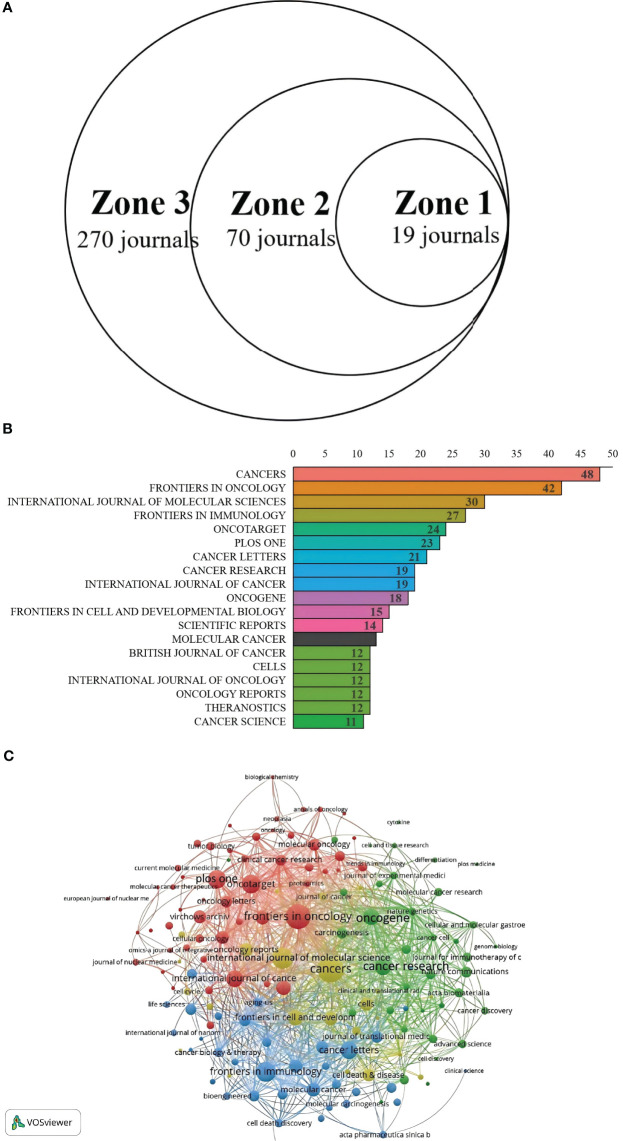
**(A)** Journal productivity analysis according to Bradford’s law. **(B)** Distribution map of journal publications in zone one. **(C)** Citation network map of journals.

Bradford’s Law has been applied in order to determine CAFs and the CRC of core journals. The literature (journals), as shown in **Figure 6A**, was divided into 3 zones. The Zones 1 and 2 each contained 19 journals. Zone 3 included 270 journals. The top 19 journals are shown in **Figure 6B**. CANCERS is ranked first (N=48), followed by FRONTIERSINONCOLOGY(N=42), the INTERNATIONALJOURNAL of MOLECULAR RESEARCH (N=30), the FRONTIERSIN IMMUNOLOGY(N=27), then ONCOTARGET. In **Table 3**, 15 of the 19 top journals by volume are in Q1 (the first quartile) in Journal Citation Reports, and 3 are in Q2 (the second quartile). A total of nine journals has an impact factor (IF) above five. INTERNATIONAL JOURNAL OF CANCER is the most cited journal among the top 19, with 275 citations, followed by ONCOGENE (228 citations) and CANCERS (151 citations). The first JCR quarter is made up of 15 journals, while the second quarter has three journals. This indicates that these are the journals most highly valued and reflect the current trends in research. **Figure 6C** shows the co-citation map, which illustrates how closely journals are related. The clustering of journals into four groups is based on the node sizes, which represent the number of co-citations. Link thickness indicates the degree of association. The journals of the same color share the same topics. Most of these are represented by the red cluster, which is centered around cancer research. This indicates the strongest theme connection between the journal and its most frequent citations.

**Table 3:** Leading journals in zone one for colorectal cancer and cancer-associated fibroblasts studies ranked by issuance and citations.

Rank	Journal title	Records	Total citations	Average citation	JCR (2025)	IF (2025)
1	CANCERS	48	151	3.15	Q1	4.5
2	FRONTIERS IN ONCOLOGY	42	132	3.14	Q2	3.5
3	INTERNATIONAL JOURNAL OF MOLECULAR SCIENCES	30	63	2.1	Q1	4.9
4	FRONTIERS IN IMMUNOLOGY	27	39	1.44	Q1	5.7
5	ONCOTARGET	24	132	5.5	–	0
6	PLOS ONE	23	114	4.96	Q1	2.9
7	CANCER LETTERS	21	109	5.19	Q1	9.1
8	INTERNATIONAL JOURNAL OF CANCER	19	275	14.47	Q1	5.7
9	CANCER RESEARCH	19	149	7.84	Q1	12.5
10	ONCOGENE	18	228	12.67	Q1	6.9
11	FRONTIERS IN CELL AND DEVELOPMENTAL BIOLOGY	15	31	2.07	Q1	4.6
12	SCIENTIFIC REPORTS	14	31	2.21	Q1	3.8
13	MOLECULAR CANCER	13	113	8.69	Q1	27.7
14	THERANOSTICS	12	141	11.75	Q1	12.4
15	BRITISH JOURNAL OF CANCER	12	72	6	Q1	6.4
16	ONCOLOGY REPORTS	12	49	4.08	Q2	3.8
17	INTERNATIONAL JOURNAL OF ONCOLOGY	12	41	3.42	Q1	4.5
18	CELLS	12	20	1.67	Q2	5.1
19	CANCER SCIENCE	11	127	11.55	Q1	4.5

### Keywords co-occurrence, clusters and bursts

**Figure 7:**
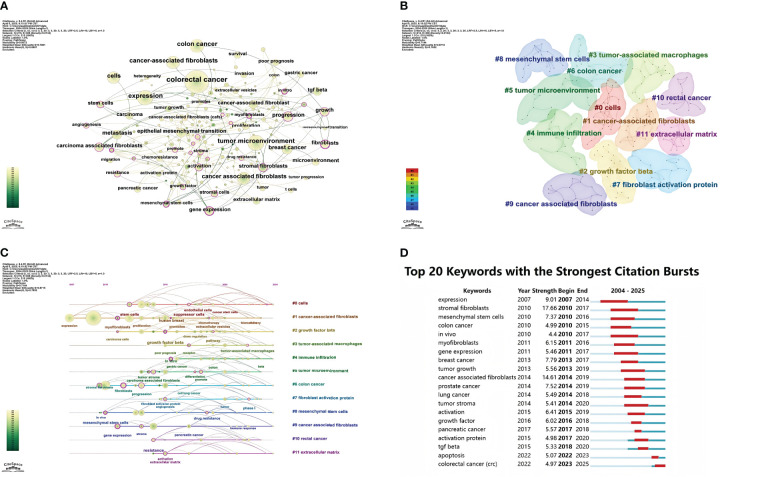
**(A)** Keyword network visualization in cancer-associated fibroblasts and colorectal cancer research. **(B)** Cluster distribution map of keywords in cancer-associated fibroblasts and colorectal cancer research. **(C)** Timeline chart of keyword clustering on cancer-associated fibroblasts and colorectal cancer. **(D)** Keyword burst chart of cancer-associated fibroblasts and colorectal cancer.

Keywords distill and summarize the essence of literary themes. Tallying keywords in research literature and identifying those with high citation counts reflects their significance, offering a lens to analyze the evolution of research hotspots (18). As depicted in **Figure 7A**, the visualization analysis conducted by CiteSpace software comprises 215 nodes, each representing a keyword extracted from 1,145 papers. Larger nodes indicate higher keyword frequency, while thicker links signify closer keyword co-occurrence. Beyond the prominent nodes “cancer-associated fibroblasts” and “colorectal cancer,” keywords like “tumor microenvironment,” “chemoresistance,” “tumor progression,” and “angiogenesis” are closely linked to CAFs and CRC. Cluster analysis of keywords enhances clarity on specific research content within the CAF and CRC domain. **Figure 7B** illustrates the visualized keyword clusters, revealing 12 clusters from #0 to #11, with smaller numbers indicating more keywords per cluster. Overlapping clusters in the keyword map signify close associations. The cluster timeline view (**Figure 7C**) displays each cluster’s time span and inter-cluster correlations, mapping research evolution with the x-axis as publication years and the y-axis as cluster numbers. This view highlights how knowledge develops over time, offering directional insights for future research. Our cluster analysis reveals two primary research focuses: (1) CAFs, a key tumor microenvironment (TME) component, secrete factors like TGF- β and PDGF to promote tumor cell proliferation, invasion, and metastasis; (2) In addition to interacting with tumor cells and other cell types, CAFs play a role in regulating the makeup and function of the TME. As per the timeline view, keywords that exploded early (2007–2015), such as “expression,” “stem cells,” and “growth factor beta,” underwent significant changes in burst incidence during the mid-stage (2015–2020), with keywords like “cancer-associated fibroblasts” and “tumor microenvironment.” From 2020 to 2024, keywords shifted markedly to include “colon cancer,” “rectal cancer,” and “extracellular matrix.” **Figure 7D** details the top 20 keywords, their burst timeframes, and strengths, with higher numerical values indicating stronger citation bursts. Red bars denote burst periods, and blue bars non-burst periods. During the early burst phase (2007–2015), keywords like “expression” and “stromal fibroblasts” had high burst intensity. In the mid-stage burst (2015–2020), keywords such as “tumor stroma” and “activation” were concentrated on CAFs’ role in the TME. Recently (2020–2025), keywords like “colorectal cancer” and “apoptosis” have shown high burst intensity, focusing on CAFs’ specific role in CRC and their relation to apoptosis.

## Discussion

The largest population of stromal cells in TME is CAFs. Not only do they alter TME immunesuppressive properties but also affect immune cell function, and aggregate via secreted factors, helping tumor cells to escape the immune system (19, 20). The Web of Science (WOS)’s publication data was used to conduct a bibliometric analysis on CAFs in relation to CRC. This study aims to determine the most important research and development areas for CAFs in CRC.

### General distribution

Global publications are increasing steadily, indicating a great deal of interest in the field. This suggests a huge potential for research. There are differences between the publication volume and citation count from a country/regional perspective. China is the leader in terms of publication volume, but it trails behind the United States when it comes to total and average counts. There are four reasons: The research of some Chinese CRCs and CAFs is more descriptive and basic, and lacks in-depth exploration and novel therapeutic strategies. U.S. Studies, on the other hand, often combine basic research with clinical applications, and emphasize translational medicine, innovative therapies, and therapy development. This is why they receive more international attention, and are cited. II. Certain Chinese studies can have shortcomings in their methodological design, such as small sample sizes and less rigorous experimental designs, or simple statistical methods. This will reduce the likelihood of citations. U.S. Studies usually use stricter design and advanced techniques to provide stronger evidence. They also attract more citations. III. In CRCs and CAFs, the U.S. works closely with other countries to improve research impact and quality. IV. Researchers in the U.S. have a greater history of CRC research and CAFs, which gives them an advantage. China has made some progress but it is still a long way behind. On the bright side, four of the top five research institutions are in China, highlighting its important influence in this field. China must now improve research quality, increase investment in basic research, and adopt stricter designs and advanced techniques like multi-omics sequencing, single-cell RNA sequencing, and spatial transcriptomics to strengthen evidence. Encouraging interdisciplinary collaboration-for instance, between oncology, immunology, and bioinformatics-to study CAFs in CRC from diverse angles is also crucial. Furthermore, building relationships with top international research teams for joint projects, resource, and data sharing can elevate research standards and impact. Finally, active participation in international academic conferences and exchanges will help Chinese researchers stay updated on global trends, showcase their work, and enhance China’s international visibility in CRC and CAFs research. Sun Yat-sen University leads globally in publications on colorectal cancer (CRC) and cancer-associated fibroblasts (CAFs). Recent studies indicate that CAFs boost PD-L1 glycosylation through the glycosyltransferase EDEM3, thereby facilitating immune evasion. EDEM3, which is highly expressed, recruits’ macrophages based on glucose metabolism. This study shows that restricting the glucose metabolism, as with a 2-DG and fasting-like diet, could work in conjunction with PD-1 antibody to increase antitumor immune response and overcome resistance against PD-1/PDL1 blocking (21). Our analysis of the most influential authors shows that De Wever, Olivier has published 16 relevant research papers with a total of 1,409 citations, making him the most prolific and most cited author in publications related to CRC and CAFs. His latest research reveals that EVs from epithelial CRC cells, which are rich in miR-200, can inhibit the transformation of fibroblasts into myofibroblasts. In contrast, EVs from mesenchymal CRC cells, which have low levels of miR-200, cannot suppress this transformation. The study also indicates that miR-200 regulates fibroblast phenotype by targeting ZEB1, and in human CRC samples, miR-200 shows a negative correlation with CAFs markers. The findings provide new insight into CAFs and the role they play in CRC (22). Marc Bracke has become the second-most influential author with his paper titled, “Glucocorticoids decrease colon cancer cells proliferation and invasion through effects on cancer associated fibroblasts.” The study examines the modulation of cancer-associated fibroblasts by glucocorticoids to suppress colon cancer (CRC), cell invasion and proliferation, as well as enhance cancer cell migration. Upon treating CAFs with dexamethasone, the team noted decreased secretion of specific factors, including tenascin C and hepatocyte-growth factor (HGF). The conditioned media from CAFs treated with Dex (CMDEX), inhibits CRC cells proliferation and invasion significantly compared to CAFs that were not treated (CMCTRL). *In vivo*, using a chick chorioallantoic membrane (CAM) model, tumors formed from Dex-treated CAFs and CRC cells showed reduced formation and invasion. These findings suggest GCs alter CAF secretions to influence CRC cell behavior, offering a novel therapeutic perspective (23). Cristina Peña has become the third most influential author in this field. Her latest paper, “Cancer-associated fibroblast-derived gene signatures determine prognosis in colon cancer patients,” reveals that CAFs’ genetic traits impact CRC patient prognosis. By comparing CAFs and NFs, researchers found a 596-gene “CAF signature” linked to cell metabolism and protein binding. Analyzing 1235 colon cancer patients, those with a high “signature gene score” had shorter overall survival. Research also revealed that ncRNAs within exosomes derived from CAFs significantly influence tumor cell proliferation and DNA repair, underscoring the importance of CAFs and their exosomes as prognostic biomarkers in CRC (24). Moreover, CANCERS is the top journal for publishing articles on CRC and CAFs, with most papers centering on molecular mechanisms. Recent studies have shown that fibroblast activity protein (FAP), which is expressed highly in many CRC tissues, but not detected in normal tissue, could be a potential target for antibody-conjugate therapy. CRCs that express high levels of PDPN and MMP2 also show immune-related characteristics, suggesting that CAF markers could be used as targets in immune engagement therapy. Overall, this research underscores CAF proteins’ potential as novel anticancer therapeutic targets and biomarkers (25). Notably, analyses of international cooperation, as well as collaboration networks among research institutions and authors, reveal sparse collaboration density and limited interaction. Thus, enhancing academic collaborations across countries and institutions to form a unified academic community is crucial for advancing clinical and academic research in this field.

The analysis of co-occurrences, clusters, and changes in timelines shows the evolution and depth of research into CRCs and CAFs. In the years 2004-2010, research focused on “CAFs”, CRC”, “stroma” and “tumor Microenvironment”. Research aims to clarify CAFs’ role in CRC and their relationship with the tumor microenvironment. CAFs form the main stromal cells in tumor environments. By secreting growth factors and remodeling the extracellular matrix, they have an important impact on CRC growth and progression. These studies provided the theoretical foundation for future research. They also highlighted CAFs’ importance in CRC progression and provided a framework to explore their functions and mechanisms. It was at this stage that CAFs were systematically integrated into CRC studies for the first time. The role of CAFs within tumor microenvironments was defined and the concepts and mechanisms for future research were established. The CAFs that secrete growth factors accounted for part of tumor growth and offered potential targets for CAF-targeted therapy. Extracellular matrix remodeling also helps predict cancer invasion. The knowledge gained was helpful in developing diagnostic and therapeutic approaches (26).

Research between 2011 and 2020 highlighted CAFs’ key role in CRC’s “chemoresistance”, “tumor progress”, and “angiogenesis”. Researchers found that CAFs increase CRC cell chemoresistance through secreting proteins and activating signaling pathways. This affects treatment outcomes (27). The CAFs also promote the progression of tumors by increasing CRC cell invasiveness, and their ability to metastatically spread (28). In angiogenesis, CAFs secrete angiogenic factors like VEGF. It stimulates new blood vessel formation for metastatic growth of cancer (29). These findings offered new insight into CAF’s complex role in cancer progression and suggested possible targets to overcome chemoresistance. This research phase is important for several reasons. In chemoresistance studies, the CAFs ability to increase CRC cell drug resistance through protein secretion or signaling pathways activation is a major factor in poor chemotherapy outcomes. CAFs have been found to activate pathways such as NF-kB in order to enhance CRC cell resistance to oxidative stresses and chemotherapy-induced apoptosis. This has led to drug development that targets CAF-related pathways (30). In the second, tumor progression research is exploring CAFs’ ability to induce EMT. This could lead to new ways of controlling cancer invasion and metastatic spread. As a crucial step in CRC progression and distant metastasis, EMT can be triggered by CAFs secreting cytokines like TGF-b and TNF-a, promoting the epithelial-to-mesenchymal shift in CRC cells (31). It also clarifies molecular mechanisms of cancer invasion and metastasis, and identifies potential anti-EMT targets such as blocking TGF-b pathways to reduce CRC cell invasion. CAFs secreting angiogenic factor provides an alternative approach to anti-angiogenic therapy. CAFs promote tumor angiogenesis through factors such as VEGF A and VEGF C, supporting nutrition of tumors and metastasis (32, 33). Researchers have created monoclonal antibody and small molecule inhibitors that target the VEGF path. Bevacizumab has proven to be effective in CRC treatment. This gives patients new hope (34).

**Figure 8:**
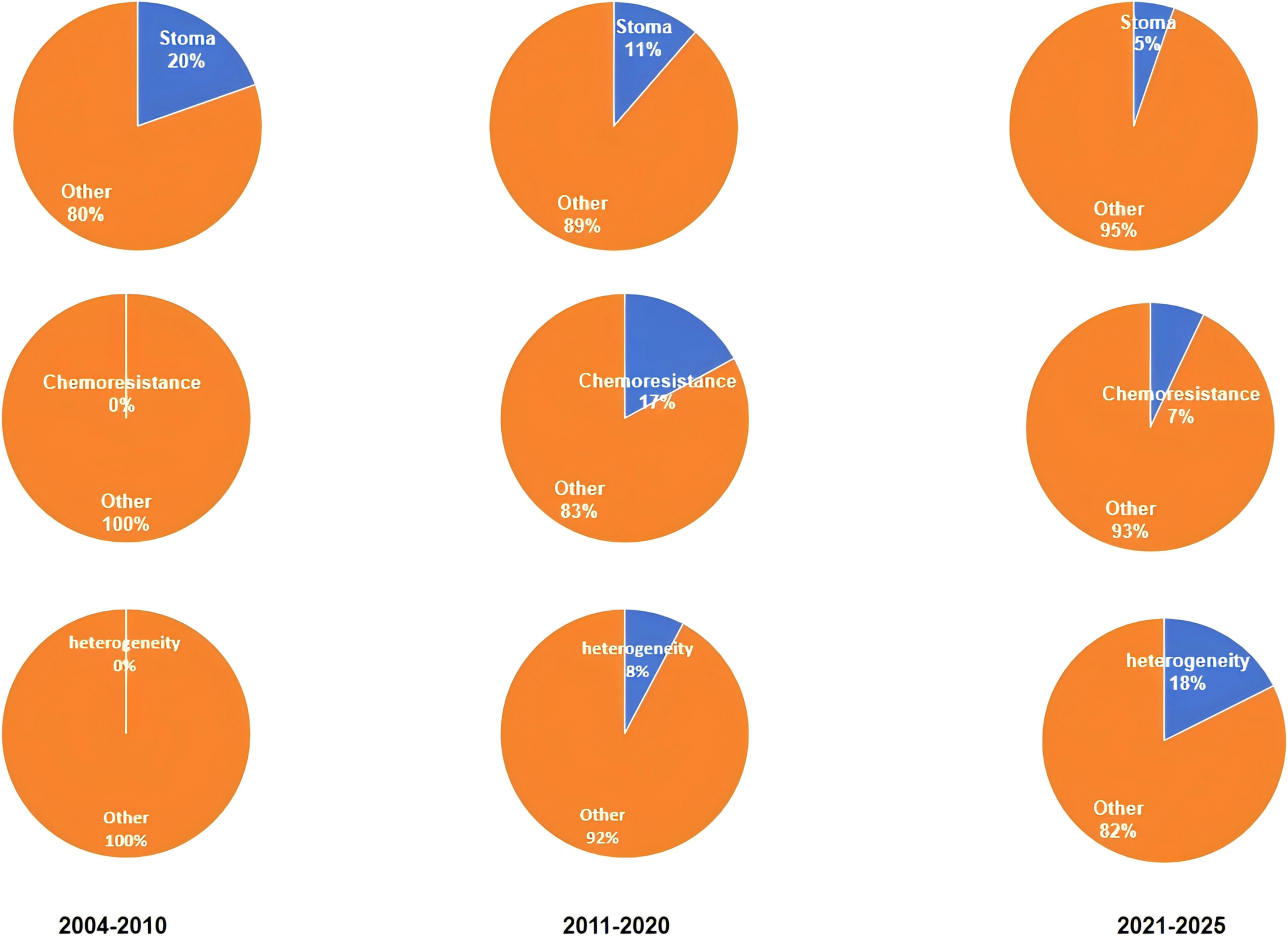
The co-occurrence percentages of the keywords stoma, chemoresistance, and heterogeneity.

Recent research (between 2021-2025), has been focused on the “heterogeneity”, “immune-evasion” and “targeted treatments” of CAFs. According to research, CAFs have a highly heterogeneous origin, phenotype, and functional characteristics. It is possible that the heterogeneity in CAFs explains their different roles during CRC treatment and development. CAFs might release immunosuppressive substances like transforming-growth-factor-b (TGF-β) and interleukin-10, potentially suppressing immune cell function and aiding tumor immune evasion in CRC (35). Based on these findings, researchers have begun to explore targeted therapies against CAFs They aim to develop new treatments by targeting CAFs’ specific signaling pathways or markers. It could improve CRC patient survival and treatment. This phase of research is crucial for CRCs and CAFs. First, CAFs’ heterogeneity was discovered. This is a significant breakthrough. This discovery has changed our understanding of CAFs. The research has shown that CAF heterogeneity is not just reflected by the different cell-surface marks, but also their diverse functional properties. Some CAFs, for example, promote tumor cell adhesion by secreting extracellular matrices proteins. Others inhibit the antitumor immunity by secreting immune modulatory factors (36, 37). It is because of this heterogeneity that single treatment strategies for CAFs are often ineffective. A detailed study of subtypes of CAFs, as well as their functional characteristics can be used to develop better treatment plans. This can help to design personalized treatment plans for the different CAF subtypes and improve the treatment effectiveness. Second, new immunotherapy insights have been gained from the study of mechanisms that CAFs use to evade CRC immunity. The CAFs secreted immunosuppressive agents such as TGF-β and IL-10 that suppress the proliferation and activity of T-cells while promoting differentiation of regulatory T-cells (Tregs). This creates an immune-suppressive microenvironment. The discovery of a novel mechanism for CRC immuno evasion provides new targets in immunotherapy. Blocking the pathways by which CAFs release TGF-β should enhance T-cells-mediated antitumor immunity and increase the effectiveness of immune checkpoints inhibitors, such as PD-1 and CTLA-4 inhibitors (38). The exploration of targeted treatments against CAFs brings new hope to personalized CRC treatment. Researchers have created a number of drugs that target CAFs based on their deep understanding of its functions and mechanisms. Researchers have developed monoclonal antibodies that target CAF markers like fibroblast activation protein (FAP) and a smooth muscle actin. These antibodies can target CAFs by binding to markers specifically (39, 40). In preclinical studies, small-molecule inhibitors targeting signaling pathways that are linked to CAFs like TGF-β inhibitors have demonstrated promising efficacy (41, 42). These targeted therapies offer more personalized options in the treatment of CRC. The treatment plan can then be more accurately tailored based on tumor characteristics and CAF phenotypes. This will improve the quality of life and survival rates of the patients. This study selected randomly a word from each phase. We then used VOSviewer to analyze the frequency with which each keyword appeared. This analysis enabled us to determine the proportion and frequency of keywords in various phases. It also helped quantify research hotspots at each phase. This study’s results can be seen in **Figure 8**. It confirms that the clustering of literature results is accurate.

In general, the research into CRCs and CAFs is moving away from simply describing their biological properties to examining their role as dynamic cells in tumor microenvironments and their interaction with other cells such immune cells. The single-cell technology advances that have enabled researchers to explore CAF heterogeneity and specific functions are partly responsible for this shift. Researchers are also more interested now that CRC is being treated with targeted therapies and immunotherapy.

Further research should be done on CAFs and CRC. Further research is needed to determine the specific role of CAFs and their functional heterogeneity in various tumor microenvironments, including tumor invasion, drug resistance, and metastasis. It is important to investigate how CAFs influence immune cell functions through exosomes or secreted factors, and promote tumor immune evasion. Another important direction is to develop targeted strategies for CAFs in order to combat tumor drug resistance, and improve the efficacy of immunotherapy. It is also important to identify intervention strategies that can be used in order to study how CAFs regulate signaling pathways and promote drug resistance. Ultimately, more in-depth research into CAFs’ molecular mechanisms, such as non-coding RNAs and exosomes, and their interactions within the tumor microenvironment will lay the foundation for transforming basic research findings into clinical applications.

### Frontier topics

#### 1 CAFs’ Significant heterogeneity

**Figure 9:**
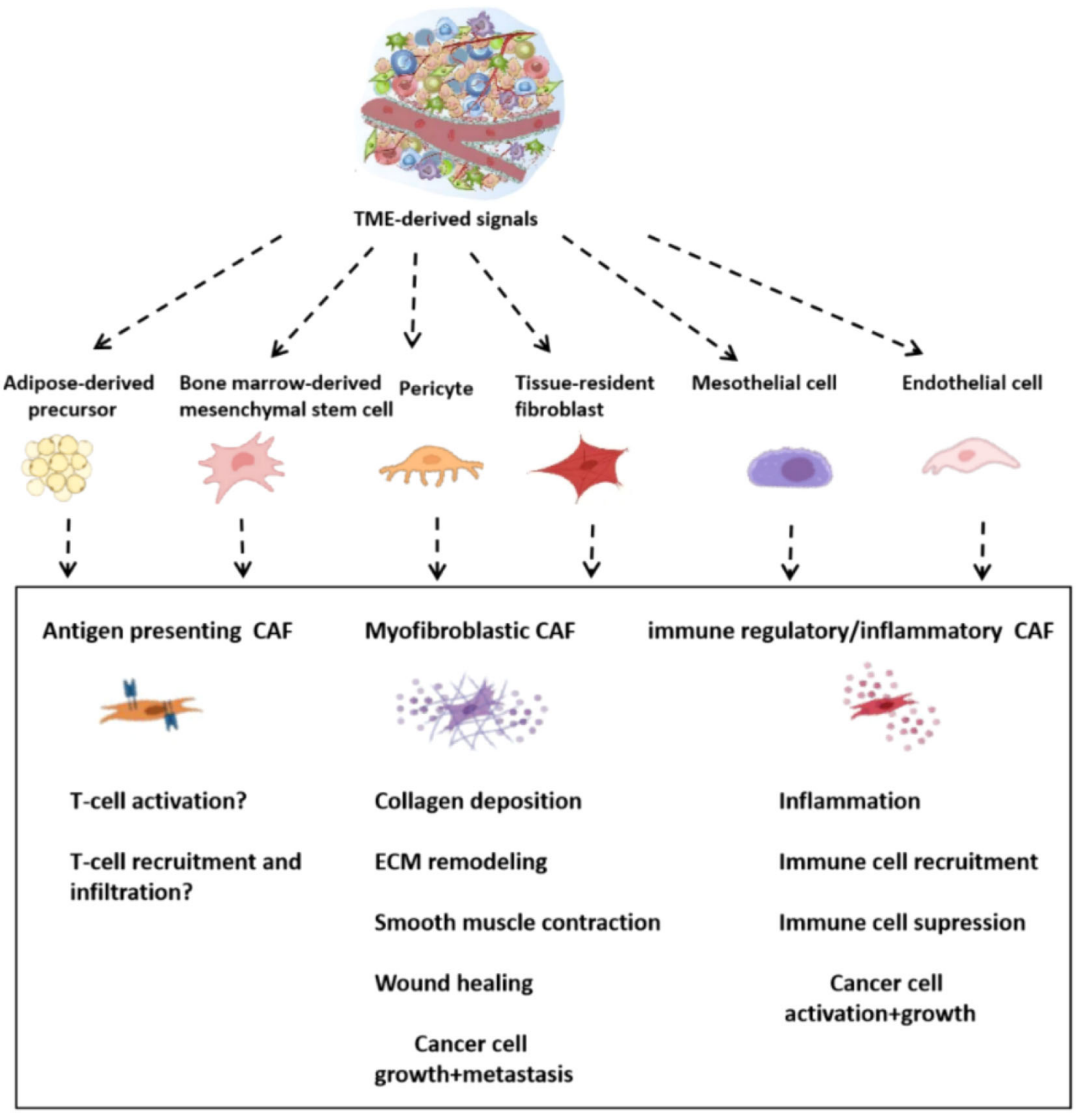
Primary CAF subsets and their potential origins.

The CAFs are highly heterogeneous in terms of their origins and functions, phenotypes (43). The TME can be formed by adipocytes or endothelial or mesenchymal stem cells that have been recruited to it from the bone marrow, as well as precursor cells derived from pericytes, adipocytes or mesenchymal stem cells (44–46) (**Figure 9**). It has been established, for example, that immune regulatory fibroblasts are able to modulate the immune response and actively participate in tissue immune regulation. The immune-regulating CAFs are a subset of the cancer-associated fibroblasts. By secreting cytokines, they influence immune cell activity, proliferation and migration. They become important components of the microenvironment that surrounds tumors, and can promote or suppress tumor growth (47). The heterogeneity of the fibroblasts is evident in their diverse functions in cancer progression and metastasis. These include promoting tumor cell growth, enhancing angiogenesis and remodeling ECM. Different CAF subsets also exert different impacts on the progression of tumors. Some CAFs may stimulate the growth of tumors, while other can inhibit it. Growing evidence suggests that some subsets of cancer-associated fibrocytes can suppress tumors (48, 49). Meflin is a GPI-anchored glycosylphosphatidyl-inositol-anchored (GPI-anchor) protein that serves as a tumor-restraining marker in pancreatic ductal adenocarcinoma (50, 51). Meflin can also be found in mesenchymal stem/stromal cell undifferentiated cells. It is downregulated either by TGF-β or stiffness of the substrate. It is therefore likely that the loss of Meflin correlates with tumor-promoting CAF traits (52). Myofibroblasts are activated fibroblasts that have a high production of extracellular matrix (44). In mice with PDAC caused by spontaneous mutations, deletion of Col1A1 from a-SMA positive myofibroblasts increased cancer progression (53). Col1A1 was not deleted in stromal cell Fsp1 positive cells, but this did affect the disease progress or collagen deposition. In a-SMA positive myofibroblasts, deletion of Col1A1 led to changes in the immune system including an increase in myeloid suppressor cells and fewer lymphocytes. The cancer cell chemoattractant Cxcl5, which is a powerful MDSC chemoattractant, was increased due to the reduced type I collagen in tumor stroma (53). The modulation of the immune system by tumors can be achieved by collagen produced by CAFs. Recently, studies have revealed the double roles that CAFs play. Based on single cell RNA-seq, CAFs derived from hepatic stellate cells in desmoplastic liver metastatic mouse models were classified into myCAFs, iCAFs, and mesCAFs (54). The tumor-restricting effects of type I collagen are highlighted by the fact that selective deletion in CAFs increases metastatic tumour growth. Interestingly, hyaluronan and HGF were tumor-promoting in myCAFs. Has2 and col1a1 are mainly expressed by myCAFs. This indicates that myCAFs play opposing roles in different contexts (54). The gastrointestinal tract is affected by the transcription factor Forkhead Box L1 (FOXL1) expressed during organogenesis in the anterior mesenchyme of gut (55). The intestinal stem cells niche is formed by subepithelial fibrocytes that are FOXL1 positive (56). The BMP signals control epithelial differentiation and regeneration of the alveolus (57).In lung fibroblasts, FOXL1 controls BMP2 and BMP4 ligands and their antagonists such as GREM1 (58). The expression of FOXL1 in subepithelial or intestinal stem cells may contribute to CRC (59).The current research does not determine whether there are distinct CAF subtypes that play tumor-promoting and -suppressive roles. It also doesn’t know if CAFs have the ability to switch roles or secrete both pro-tumorigenic as well anti-tumorigenic agents. To improve our understanding of the CRC, and potentially develop novel therapies for CRCs, we need to investigate CAF heterogeneity.

#### 2 CAFs and tumor proliferation and migration

**Figure 10:**
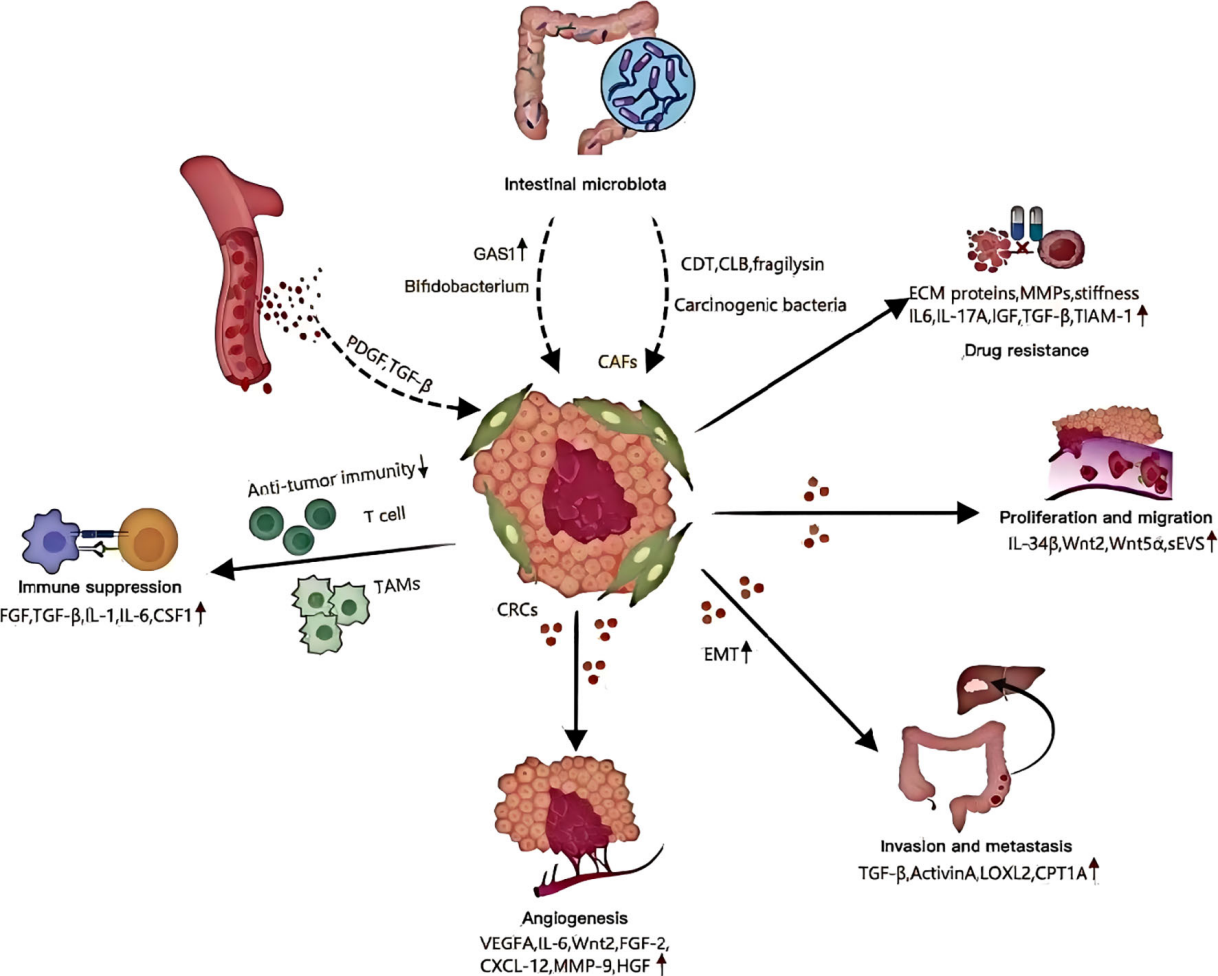
Roles of cancer-associated fibroblasts.

Recently, studies revealed that even before the malignant transformation of fibroblasts (60), they may display pro-tumorigenic properties. To explore the colorectal cancer pathogenesis, researchers isolated CAFs (cells of fibroblasts) and NFs (nuclear fibroblasts) from fresh surgical samples taken from patients with colorectal carcinomas. Co-culturing CRC cells with CAFs showed significantly enhanced proliferative and migratory capabilities (61). It is believed that CAFs promote CRC proliferation and migration by secreting circular RNAs, lncRNAs (long noncoding RNA), microRNAs as well as a variety of other cytokines (62) (**Figure 10**). PD-1, and its ligand, PDL1, interact to negatively regulate T-cells activation. They also influence tumor growth and apoptosis. Zhang and his colleagues carried out research. CAFs increased PD-L1 phosphorylation as well as expression. This could be because CAFs suppress cytotoxic T cell proliferation through the ERK5/PDL1 axis. It would promote cancer growth and reduce the immune system’s inhibition (63). Additionally, Hirashima et al. In CRC tissues, the expression of CAFs was compared with that of NFs. The gene sets that are associated with Wnt pathways were found to be highly expressed in colorectal CAFs. CAFs activated Wnt pathway by secreting Wnt2 or recombinant Wnt5a. This resulted in a substantial rise in cancerous cell proliferation (64). Synergistic interactions of tumor-associated macrophages with CAFs increased CRC cell movement, suggesting that different immune cells within the microenvironment are crucial in regulating CAFs’ biological functions (6).

#### 3 CAFs and tumor immune suppression

By regulating the recruitment and differentiation of immune cells (65), the intricate interplay between CAFs and immune cells can lead to tumor immune evasion (**Figure 10**). MSCs, which are precursors to CAFs (colorectal carcinomas), increase tumor growth through the immune system’s inhibitory effects. One mechanism MSCs/CAFs employ to control the exhaustion T cells is sialylation. By targeting sialylation of stromal cell, it may be possible to overcome the immune suppression that CRC tumors experience (66). Yang and his colleagues published research in this area. Researchers found that CAFs with BCL9 gene loss have a pro-tumor effect, resulting in abnormal activation of the Wnt/β Catenin pathway. The T-cells are then unable to provide antitumor immune responses (67). Tumor-associated macrophages play a role in all cancer types. When colony stimulating factors 1 is released by cancerous cells, CAFs can express less granulocyte-specific chemokines. This interaction is disrupted by the treatment of colony-stimulating factor 1 receptor inhibitors, which results in increased granulocyte recruitment to tumors (68). In clinical trials, colony stimulating factor 1 receptor inhibits were used to target tumor-associated macrophages, such as intestinal cancers. However, their effectiveness was limited (69). The CAFs are able to counteract these therapeutic agents’ effects by recruiting myeloid suppressor cells derived from polymorphonuclear mononuclear cells at tumor sites. This results in an immunosuppressive tissue microenvironment (TME) (68). The involvement of pentraxin 3, in the context of CAF mediated immune suppression promotes M2-like macrophage polarization. Pentraxin-3 expression in CRC patients is negatively correlated with the fibroblasts, inflammatory signals and poor survival outcome (70). CAFs could potentially modulate the cytotoxic function of T-lymphocytes, which may help tumor cells avoid detection and attack by the immune system (71). It is widely acknowledged that CAFs release cytokines and chemokines, which interact with immune cells present in the TME. The interaction alters the transformation of T-cell phenotypes, and also activates the natural killer (NK) cell functions. It also regulates the M2 macrophage phenotype, contributing to the immunosuppression in the tumor microenvironment (72–75).Chen et al. The NIH-3T3/Src cell line was stably expressing Src, a proto-oncogene tyrosine kinase. Researchers found that the conditioned medium from this cell line induces M2 and Ana-1 in bone-marrow macrophages, which are known to promote CRC. Research also showed that IL-6 produced by NIH-3T3/Src cells plays a critical role in M2 polarization and tumorigenicity. It may be possible to develop new strategies for CRC treatments based on this information. The CAFs of patients with CRC were shown to increase the expression levels of the vascular cells adhesion molecules-1. This increased monocyte adhesion. By secreting IL-8, CAFs induce macrophages into M2 polarization. In collaboration with CAFs, this process suppresses the activation of NK cells and their ability to kill CRC cells (72). This study provides a fresh perspective on the synergistic role of CAFs in CRC immune evasion. The study also identifies a potential target for blocking CRC immunity. Our comprehension of CAFs’ role in tumor immune surveillance remains in its infancy, despite growing interest in cancer immunotherapy. Research into CAFs is needed to find promising target molecules and CAF subsets. Innovative treatments can be developed to enhance clinical immunotherapy.

#### 4 CAFs and tumor angiogenesis

The angiogenesis process is critical in different stages of cancer progression. It also plays a major role in the growth and invasion of colorectal carcinoma (CRC). The importance of angiogenesis lies in the abundance of blood vessels which supply oxygen and nutrients to tumor tissues. Angiogenesis, a malignant tumor hallmark, is linked with tumor growth, metastasis and the patient’s prognosis. Angiogenesis is triggered by vascular endothelial-growth factor (VEGF), the strongest proangiogenic agent. VEGF stimulates angiogenesis through binding to the receptor VEGF-2 (VEGFR2) found on endothelial cell surface (76–79). The stromal cell population is also involved in the regulation of neovascularization (80). Cancer-associated fibroblasts directly stimulate tumor angiogenesis through the release of pro-angiogenic factor and indirectly via production of extracellular matrix. Cancer cells produce minimal VEGF in CRC. CAFs, however, are a major source of VEGFA via the release of interleukin-6 (80, 81). This complex interaction between colon cancer and tumor microenvironment facilitates transformation of normal fibroblasts into CAFs by modulating IL-6, enhancing VEGF release and promoting tumor growth (82). Additionally, Unterleuthner et al. WNT2 produced by CAFs is crucial in the promotion of blood vessel formation in CRC. The angiogenic capacity of tumor vessels is significantly reduced when WNT2 is silenced in CAFs. This highlights that increased WNT2 expression by CAFs makes tumors more susceptible for invasion and metastasis (83). CAFs also promote tumor formation by secreting factors that increase angiogenesis. These include VEGF, CXC motif-chemokine-ligand-12, matrix metalloproteinase-9 and hepatocyte-growth factor (HGF) (84). The permeable vessels within tumors lead to the release of proangiogenic growth factors, such as PDGF and TGF-β, through degranulation (85). This further activates fibroblasts. These studies collectively provide compelling evidence for CAFs to be considered as an effective therapeutic target in order to alter tumor vasculature.

#### 5 CAFs promote tumor invasion and metastasis

They are important players in tumorigenesis. They promote metastasis (86, 87) and tumor invasion. CAFs, which are an important component of TME, influence the behavior of cancer cells through various mechanisms. Their role in inducing epithelial-mesenchymal transition (EMT) is particularly significant, as this process equips cancer cells with the characteristics necessary for invasion and metastasis. TGF-β, activin A and colonic-stromal cells increase colonic epithelial migration and EMT. This increases the metastatic potential of CRC (88)(**Figure 10**). CAFs can promote EMT by secreting factors such as lysyl oxidase-2 that stimulate focal adhesion pathways (89).The process of invasion and metastatic spread could be reduced by inhibiting this. This is because CAFs control the secretion of C-C motif-ligand 2, as well as TGF-β, through regulatory elements like myosin light chain 9, to form the TME. It can affect CRC invasion (90). The CAFs also have an important impact on CRC metabolism. It increases their capacity to spread and invade. By upregulating key factors such as carnitine palmitoyl-transferase 1A (91)(**Figure 10**), CAFs promote fatty acid oxidation while minimizing glycolysis, creating a favorable environment for tumor growth and invasion. The expression of WNT2 in CAFs is associated with the invasion of cancer cells. This reveals a new aspect of CAFs’ role in metastasis (92). CAFs further drive cancer cell proliferation and invasion by secreting signaling molecules like TGF-β, leukemia inhibitory factor, and hepatocyte growth factor (HGF) (93).In particular, TGF-β induces EMT via paracrine signals, which endows premalignant cell with mesenchymal characteristics that promote invasion and metastasis (94). Additionally, CAFs are crucial in colonizing distant organs in metastasis. They do this by either creating an environment that is supportive for cancer cells, or accompanying them on their migration (95). It is essential to study factors that encourage invasion and metastasis from the perspective of EMT, metabolism and other aspects. Moreover, the effect of EMT in cancer cells on subtypes of CAFs is also important. Studies have shown that CAFs and EMT transcription factor expression are directly correlated (96). Research by Franci and al. Snail was found to be expressed predominantly in the fibroblasts surrounding tumor cells of CRC (97). CRCs exhibiting mesenchymal expression characteristics are more likely to develop distant metastasis. This is marked by an accumulation of CAFs within the stromal milieu. In addition, epithelial cancer fibroblasts have higher miR-200 levels and lower actin alpha 2, fibronectin 1, and fibronectin 2 than their mesenchymal equivalents. The discovery of a new mechanism for CRC-CAF heterogeneity elucidates how miRNA transfers via extracellular membranes contribute to this phenomenon. It also provides insights as to why CRCs that have enhanced metastatic capacity are characterized by an abundance of CAFs.

#### 6 CAFs and tumor drug resistance

Recent studies show that tumor microenvironment plays a critical role in shaping tumor cell resistance. CAFs play a key role in the development of resistance to drugs in many cancers, including CRC (98). This complex interplay involves the production of extracellular matrix (ECM) proteins and matrix-remodeling matrix metalloproteinases (MMPs) by CAFs, which not only form a physical barrier but also increase matrix stiffness and interstitial pressure, thereby hindering the effective penetration of chemotherapeutic and targeted therapies and significantly contributing to drug resistance (45, 99, 100)(**Figure 10**). It is also important to note that key soluble factors, such as interleukin-6(IL-6), interleukin-17A(IL-17A),and insulin-like-growth factor (IGF),secreted by the CAFs play a critical role in promoting chemoresistance, and amplifying cytokine release post-treatment (101, 102).The CAFs play a role not only in the physical and soluble state, but also when it comes to immune suppression. The accumulation of abnormal ECM can worsen immune suppression, and decrease the efficacy of immune checkpoint inhibitors. The involvement of TGF-β in the context of immune therapy resistance is particularly noteworthy. Blocking TGF-β with immunotherapy against anti-programmed-death protein/programmed-death ligand showed synergistic responses in a murine model of liver metastasis (100, 103). The composite stromal score system can be used to predict the radiotherapy resistance of patients with rectal carcinoma (104). When molecularly targeted medications are used, CAFs can release IGF2, HGF and hepatocyte growth factor (105). It has been shown that simultaneous inhibition of the insulin receptors, IGF1 and MET receptors as well as the epidermal growth factor receptors (EGFR) enhances efficacy of EGFR-inhibitors in colon cancer xenografts (106). In CRCs CAFs have a complex action by secreting IL-17A, which binds with the IL-17A receptor on cancer stem cell. It maintains stem-like properties in CSPDCs and increases the expression of nuclear factor kB, which induces resistance within cancer cells. The exosomes produced by CAFs can also increase stemness and promote resistance to chemotherapy in CRC. The exosomal miR-625-3p interacts with eukaryotic initiate factor 4AIII and inhibits CELF2/WWOX. This highlights the many mechanisms through which CAFs may potentiate drug resistance (107, 108). In addition, CAFs promote chemoresistance by causing the overexpression T-lymphoma invade and metastasis inducing protein-1 in CRC cell lines, making it a potential therapeutic target (109). In a co-culture setting, CAF-conditioned medium induces the overexpression of the RBCK1 gene, enhancing the stemness and resistance of CRC cells (110). This intricate web of interactions underscores the significant and diverse roles of CAFs in driving drug resistance in CRC, urging further exploration for targeted therapeutic interventions.

#### 7 Relationship between CAFs and intestinal microbiota

The intestinal microbiota is a major component of the barrier in the intestine. It actively contributes to intestinal functions and creates an environment that’s favorable for the cells. The microbial group is implicated in the various stages of colorectal carcinoma (CRC), ranging from polyps to metastasis and *in situ* growth. In 2018, the concept “carcinogenic bacteria” was introduced, which includes Escherichia, Fusobacterium, and enterotoxigenic Bacteroides fragileis. This highlights their crucial role in CRC disease pathogenesis. The tumor microenvironment is influenced by bacterial toxins such as colibactin and fragilysin. Notably, Wang Huijuan et al. Through single-cell data analysis, Wang Huijuan et al. demonstrated that the overexpression of sulfatase 1, (SULF1) in colorectal cancer CAFs is associated with a poor prognosis. Butyrate, which is produced by the gut microbiota of healthy people can also inhibit SULF1 in CAFs through targeting HDAC. Underproduction of butyrate in patients with colorectal carcinoma can increase SULF1 and CAFs. This promotes tumorigenesis (33). The interactions highlight the multiple roles of the microbiota within the intestine in orchestrating TMEs and cancer progression. The nuanced role of different intestinal bacteria is important, since some have beneficial effects for the body. For instance, Chen et al. Youth-associated Bifidobacterium activates CD143^+^CAFs, starts the Wnt pathway and increases growth arrest-specific1 expression. This results in anti-cancer effects, making it a potential therapeutic target for cancerous CRC (111). The findings highlight the dynamic interaction between intestinal microbiota and cancer cells. They provide insights into complex molecular mechanisms that shape the TME, as well as potential therapeutic avenues.

Bibliometric analyses are a powerful tool to describe the evolution of research and identify significant areas in relation between CRCs and CAFs. This method has several limitations. In the beginning, we only examined English language articles in the WOSCC data base. This approach could lead to a bias towards English publications, which would undermine the accuracy and comprehensiveness of our global analysis. We have taken several steps to reduce this bias, and ensure the validity and reliability of our study. WOSCC is a global authoritative database that includes numerous international journals of high quality. These publications attract the best research from around the world. It includes research in many different areas, despite the English publication bias. This is partly a reflection of global trends, hotspots and CRC-CAFs’ research. For the sake of greater recognition and attention, many non-English speakers also submit their work in high quality to these prestigious English language journals. We have not only considered the quantity of articles but also their influence and quality. We identified important and well-recognized research within the CRC - CAFs area by analyzing highly cited research articles, keywords in trend, key research institutions, and research authors. The findings are usually of high scientific quality and represent a wide range of research, which helps to close the gap in the field caused by English-publication bias. In future research, we will address this English-publication-bias by incorporating non-English databases such as China National Knowledge Infrastructure Database (CNKI), Japan Science and Technology Agency Database (JST), and French scientific database to include global research. We will also explore and create technologies and methods that can effectively process multilingual literature to reduce language interference, and improve the accuracy and comprehensiveness of the analysis.

We only chose original articles and reviews. Reviews provide a framework for the most recent discoveries while original articles present them. They enhance the representativeness of the study by presenting the overall field picture. The citation patterns of these studies also reflect well the citation network in this field and its knowledge flow. This provides solid support to literature metrology analysis. This selection restriction has its limitations. Other literature, such as conference papers or theses that may have valuable information, was excluded. It may result in the exclusion of important data and viewpoints, which could affect the exploration of the field. Throughout the entire research process we checked and corrected all extracted data in order to maintain its integrity and accuracy. Due to the citation delays, it is possible that some of the high-quality studies published recently have not received enough attention, requiring future updates. Our understanding of recent developments in the field may be incomplete or not timely enough. We might also miss important research that has been published recently. The findings could have a major impact on future research and knowledge diffusion, which would affect our understanding of cutting-edge dynamics. While acknowledging these limitations, our study can still effectively support researchers in grasping the progression, focal points, trends, and frontiers of CRC-CAFs research and highlighting areas for additional exploration.

In recent years, research on CRC and CAFs has garnered significant attention, with the marked increase in annual publications reflecting its growing importance. This study has identified the countries, top institutions, and researchers globally involved in CRC and CAF research. Delving into the tumor-promoting mechanisms of CAFs and developing precise targeted therapies against CAFs are current research hotspots. The long-term prognosis of patients undergoing targeted therapy may be a key focus of future research. Researchers and policymakers in this field can gain a comprehensive understanding of its evolution and frontiers.

## Data Availability

The original contributions presented in the study are included in the article/**Supplementary Material**. Further inquiries can be directed to the corresponding authors.
