# Supplementary material for: Current perspectives and trends in colorectal cancer and cancer-associated fibroblasts: a review and bibliometric analysis
Source: Front Immunol. 2025 Jul 3;16:1618742. doi: 10.3389/fimmu.2025.1618742 (PMC12267271; doi:10.3389/fimmu.2025.1618742)
Supplement: Supplementary file 1 [file DataSheet1.docx]

TI: cancer-associated fibroblasts: "cancer-associated fibroblast*" OR "tumour-associated fibroblast*" OR "tumor-associated fibroblast*" OR "tumor associated fibroblast*" OR "tumour associated fibroblast*" OR "cancer associated fibroblast*" OR "tumor-related fibroblast*" OR "tumor related fibroblast*" OR "carcinoma-associated fibroblast*" OR "carcinoma associated fibroblast*" OR "tumor associate fibroblast*" OR "cancer-associated myofibroblast*" OR "cancer associated myofibroblast*" OR "tumor-associated myofibroblast*" OR "tumor associated myofibroblast*" OR "tumour-associated myofibroblast*" OR "tumour associated myofibroblast*" OR "tumour associate fibroblast*" OR "cancer associate fibroblast*" OR "carcinoma associate fibroblast*" OR "cancer-related fibroblast*" OR "cancer related fibroblast*" OR "tumour-related fibroblast*" OR "tumour related fibroblast*" OR "carcinoma-related fibroblast*" OR "carcinoma related fibroblast*"

T2: Colorectal Cancer：“Rectal Neoplasm*" OR "Rectal Tumor*" OR "Rectal Cancer*" OR "Rectum Neoplasm*" OR "Rectum Cancer*" OR "Cancer of the Rectum" OR "Cancer of Rectum" OR "Colorectal Neoplasm*" OR "Colorectal Tumor*" OR "Colorectal Cancer*" OR "Colorectal Carcinoma*" OR "Colonic Neoplasm*" OR "Colon Neoplasm*" OR "Cancer of Colon" OR "Colon Cancer*" OR "Cancer of the Colon" OR "Colonic Cancer*"

T1 AND T2
